# SOX9 Regulates Cancer Stem-Like Properties and Metastatic Potential of Single-Walled Carbon Nanotube-Exposed Cells

**DOI:** 10.1038/s41598-017-12037-8

**Published:** 2017-09-14

**Authors:** Maria A. Voronkova, Sudjit Luanpitpong, Liying Wang Rojanasakul, Vincent Castranova, Cerasela Zoica Dinu, Heimo Riedel, Yon Rojanasakul

**Affiliations:** 1 West Virginia University Cancer Institute, West Virginia University, Morgantown, WV 26506 United States; 2 Department of Pharmaceutical Sciences, West Virginia University, Morgantown, WV 26506 United States; 3 Department of Chemical Engineering, West Virginia University, Morgantown, WV 26506 United States; 4 Department of Biochemistry, West Virginia University, Morgantown, WV 26506 United States; 5 Siriraj Center of Excellence for Stem Cell Research, Faculty of Medicine Siriraj Hospital, Mahidol University, Bangkok, 10700 Thailand; 6 Allergy and Clinical Immunology Branch, National Institute for Occupational Safety and Health, Morgantown, WV 26505 United States

## Abstract

Engineered nanomaterials hold great promise for the future development of innovative products but their adverse health effects are a major concern. Recent studies have indicated that certain nanomaterials, including carbon nanotubes (CNTs), may be carcinogenic. However, the underlying mechanisms behind their potential malignant properties remain unclear. In this study, we linked SOX9, a stem cell associated transcription factor, to the neoplastic-like properties of human lung epithelial cells chronically exposed to a low-dose of single-walled carbon nanotubes (SWCNTs). We found that SOX9 is upregulated in SWCNT-exposed cells, which is consistent with their abilities to induce tumor formation and metastasis *in vivo*. We therefore hypothesized that SOX9 overexpression may be responsible for the neoplastic-like phenotype observed in our model. Indeed, SOX9 knockdown inhibited anchorage-independent cell growth *in vitro* and lung colonization *in vivo* in a mouse xenograft model. SOX9 depletion also suppressed the formation of cancer stem-like cells (CSCs), as determined by tumor sphere formation and aldehyde dehydrogenase (ALDH) activity (Aldefluor) assays. Furthermore, SOX9 knockdown suppressed tumor metastasis and the expression of the stem cell marker ALDH1A1. Taken together, our findings provide a mechanistic insight into SWCNT-induced carcinogenesis and the role of SOX9 in CSC regulation and metastasis.

## Introduction

Engineered nanomaterials have increasingly been used for various applications, but their long-term health effects are largely unknown. Carbon nanotubes (CNTs) are one of the most commonly used engineered nanomaterials due to their unique properties such as light weight, high tensile strength, and electrical conductivity^1, 2^. However, CNTs have some negative properties as well, such as a high aspect ratio and biopersistence; therefore, questions about their potential carcinogenicity have been raised^3, 4^. Previous animal studies have shown that pulmonary exposure to single-walled carbon nanotubes (SWCNTs) induces inflammation, granulomas, and fibrosis^5, 6^, conditions that have been associated with an increased risk of lung cancer^7, 8^. In fact, some CNTs can induce or promote tumor formation in animals^3, 9–12^. Furthermore, one type of CNTs, multi-walled carbon nanotubes (MWCNTs) Mitsui-7, was classified as possibly carcinogenic to humans by the International Agency for Research on Cancer (IARC)^13^, while *in vitro* data on other CNT types were concluded insufficient to be extrapolated to humans.

We previously reported that long-term, low-dose exposure of human lung epithelial cells to SWCNTs and MWCNTs results in neoplastic-like transformation^14, 15^. Long-term treatment with CNTs was applied to mimic gradual cellular transformation during cancer development, a process that may require a prolonged exposure to carcinogens^16–18^. We also reported that chronically SWCNT-exposed cells contain a highly invasive and tumorigenic stem-like cell subpopulation^19, 20^. However, detailed information about the underlying mechanisms remains unknown.

Increasing amounts of evidence suggest that cancer stem cells or stem-like cells (CSCs), also called tumor initiating cells, are the main driving force behind tumor formation and metastasis^21, 22^. CSCs and regular stem cells share many properties, including self-renewal capacity, potency for differentiation, and resistance to apoptosis. More importantly, CSCs are typically resistant to chemotherapy and eventually give rise to recurrent tumors^22, 23^.

Many stem cell regulatory proteins are now being recognized as oncogenes because of their ability to regulate CSCs. SOX9 (SRY (sex determining region Y)-box 9) is a member of the SOX family of transcription factors, which play critical roles in embryonic development, lineage commitment, and stem cell maintenance^24^. Notably, SOX9 is involved in lung branching morphogenesis^25^, and its expression is elevated in many types of cancer, including lung, skin, brain, and pancreatic cancers^26^. In non-small cell lung cancer (NSCLC), the most common type of lung cancer, SOX9 expression highly correlates with the disease progression and poor patient survival^27, 28^. Accumulating evidence also suggests that SOX9 may regulate CSCs^29–32^. However, detailed mechanisms have yet to be elucidated. Furthermore, it is not known whether SOX9 plays a role in SWCNT-induced carcinogenesis and CSC formation.

In this study, we demonstrated that chronically SWCNT-exposed human lung cells display high levels of SOX9 expression and contain a distinct CSC subpopulation. We hypothesized that SOX9 overexpression may be responsible for the malignant phenotype observed in these cells. Consequently, we evaluated the effects of SOX9 expression on the tumorigenicity, invasiveness, and stemness of SWCNT-transformed cells *in vitro* and *in vivo*.

## Results

### Exposure to carbon nanotubes induces cell transformation

**Figure 1:**
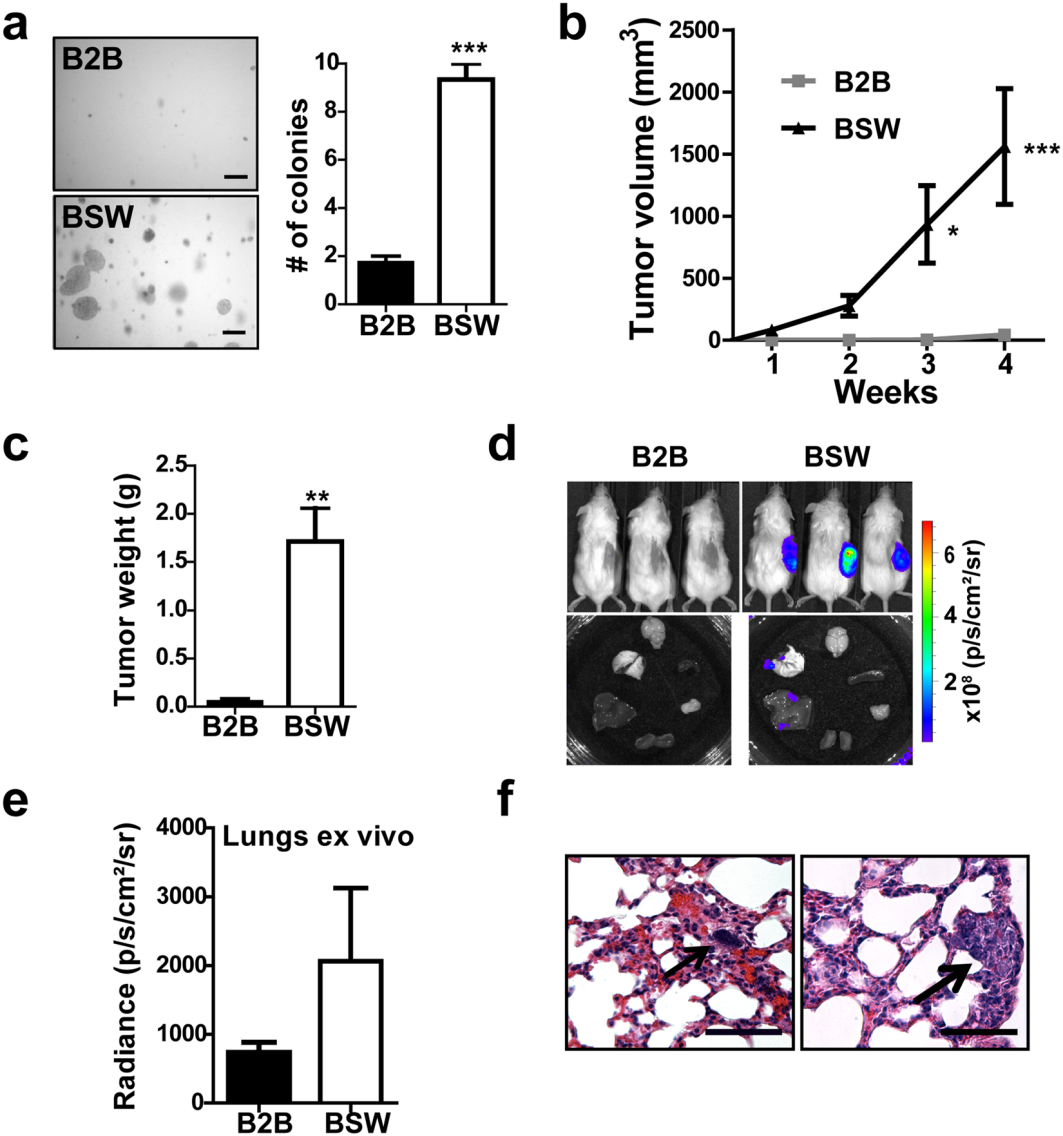
Human bronchial epithelial cells, chronically exposed to carbon nanotubes, undergo malignant transformation. (**a**) Chronically SWCNT-exposed bronchial epithelial Beas-2B (BSW) cells were cultured in soft agar for 2 weeks, and colonies over 50 μm in diameter were counted, n = 15, p < 0.0001, two-tailed t-test. Scale bar = 500 μm. (**b**) Luciferase-labeled cells were injected subcutaneously into flanks of NOD/SCID gamma mice (n = 5 per group), and tumor volume was measured weekly by an external caliper. Means are different according to Bonferroni post hoc analysis following 2-way ANOVA, p < 0.05. (**c**) Weight of isolated subcutaneous tumors at week 4, p = 0.0014, two-tailed t-test. (**d**) Top – representative bioluminescent signal from tumors at week 4, Bottom – representative luciferase signal from isolated internal organs. (**e**) Luciferase signal from lungs imaged *ex vivo*. (**f**) Representative images of H&E stained lungs from mice bearing BSW cells. Arrows point to metastatic modules. Scale bar, 50 µm. Data are mean ± SEM.

To test the potential role of SOX9 in SWCNT-induced oncogenesis, human bronchial epithelial Beas-2B cells were continuously exposed to occupationally relevant concentrations (0.02 μg/cm^2^) of SWCNTs for a period of 6 months, as previously described^14, 15, 19^. Physicochemical properties of SWCNTs used in this study are summarized in Table 1. Potential carcinogenic properties and SOX9 expression were then evaluated *in vitro* and *in vivo*. Analysis of anchorage-independent cell growth, a hallmark of cancer^33, 34^, revealed that the SWCNT-treated cells (termed BSW) formed 5-fold more colonies when compared to passage-matched control (B2B) cells (Fig. 1a). To evaluate the tumorigenic potential of BSW cells *in vivo*, these cells and the control B2B cells were genetically labeled with luciferase and injected subcutaneously into the flanks of NOD/SCID gamma mice. Tumor formation was examined over a period of 4 weeks by external caliper measurements and by bioluminescence imaging. Unlike control cells, BSW cells formed rapidly growing tumors (Fig. 1b,c). Moreover, *ex vivo* analysis at the end of the experiments showed spontaneous metastasis of the BSW cells to the mouse lungs and liver (Fig. 1d,e,f and Supplementary Fig. S1). These results indicate that SWCNT-transformed cells possess tumorigenic and metastatic properties.

**Table 1:** Physicochemical properties of SWCNTs used in this study.

Characteristic	Value	Method used for characterization
% carbon (w/w)	99%	Nitric acid dissolution, inductive coupled plasma-atomic emission spectrometry (ICP-AES, NMAM #7300)
% Fe (w/w)	0.23%	
Other metals	Not detectable	
Surface area	400–1000 m^2^/g	Nitrogen absorption-desorption technique (Brunauer Emmet Teller method, BET) using SA3100 Surface Area and Pore Size Analyzer (Beckman Coulter)
Dry length, µm	0.1–1	Field emission scanning electron microscopy
Dry width, nm	0.8–1.2

### SOX9 overexpression regulates malignant properties of BSW cells

**Figure 2:**
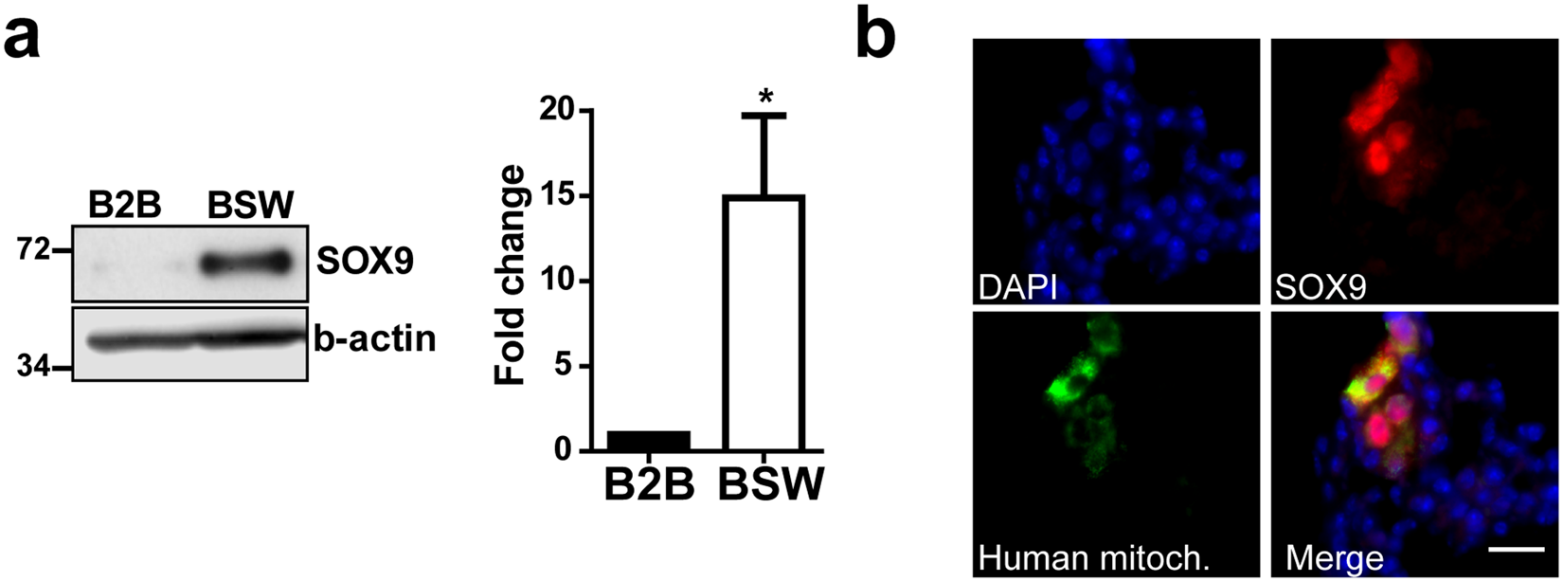
Human bronchial epithelial cells, chronically exposed to carbon nanotubes, overexpress SOX9. (**a**) Immunoblot of BSW and control cells, showing significant SOX9 overexpression in BSW cells. Right – relative SOX9 expression presented as fold change over control cells (n = 6). Data are mean ± SEM, *p = 0.0347, paired two-tailed t-test. (**b**) Representative image of lung metastases from mice bearing BSW cells subcutaneously; sections were stained with anti-SOX9 antibody and antibody against human mitochondria, nuclei stained with DAPI. Scale bar: 20 µm.

Cancer stem cells (CSCs) are considered to be the main source of cancer metastasis, chemoresistance, and tumor recurrence^21^. Previous studies have shown that SWCNT-exposed cells contain a CSC-like subpopulation identical to CSCs reported in lung cancer^19, 35^. This cell population is characterized by the expression of stem cell markers, self-renewal ability, and more importantly by chemoresistance and high tumorigenic potential^19, 20^. In this study, we examined the molecular mechanisms of CSC regulation to identify possible biomarkers and drug targets for CNT-related malignancies. SOX9, a stem cell transcription factor, has recently been implicated in CSC regulation^26, 29^ and is overexpressed in non-small cell lung cancer (NSCLC)^27, 32^. We hypothesized that SOX9 may regulate the tumorigenic and metastatic properties of SWCNT-exposed cells by controlling CSCs. Indeed, BSW cells express a high level of SOX9 protein when compared to passage-matched control cells (Fig. 2a). Furthermore, immunostaining analysis revealed that lungs of all mice in the BSW group contain SOX9 overexpressing micrometastases (Fig. 2b). Immunofluorescent staining for CSC markers in control and SWCNT-exposed cells demonstrated that CSC markers are overexpressed in BSW cells and that the CSCs are the cells that have elevated SOX9 expression (Supplementary Fig. S2).

**Figure 3:**
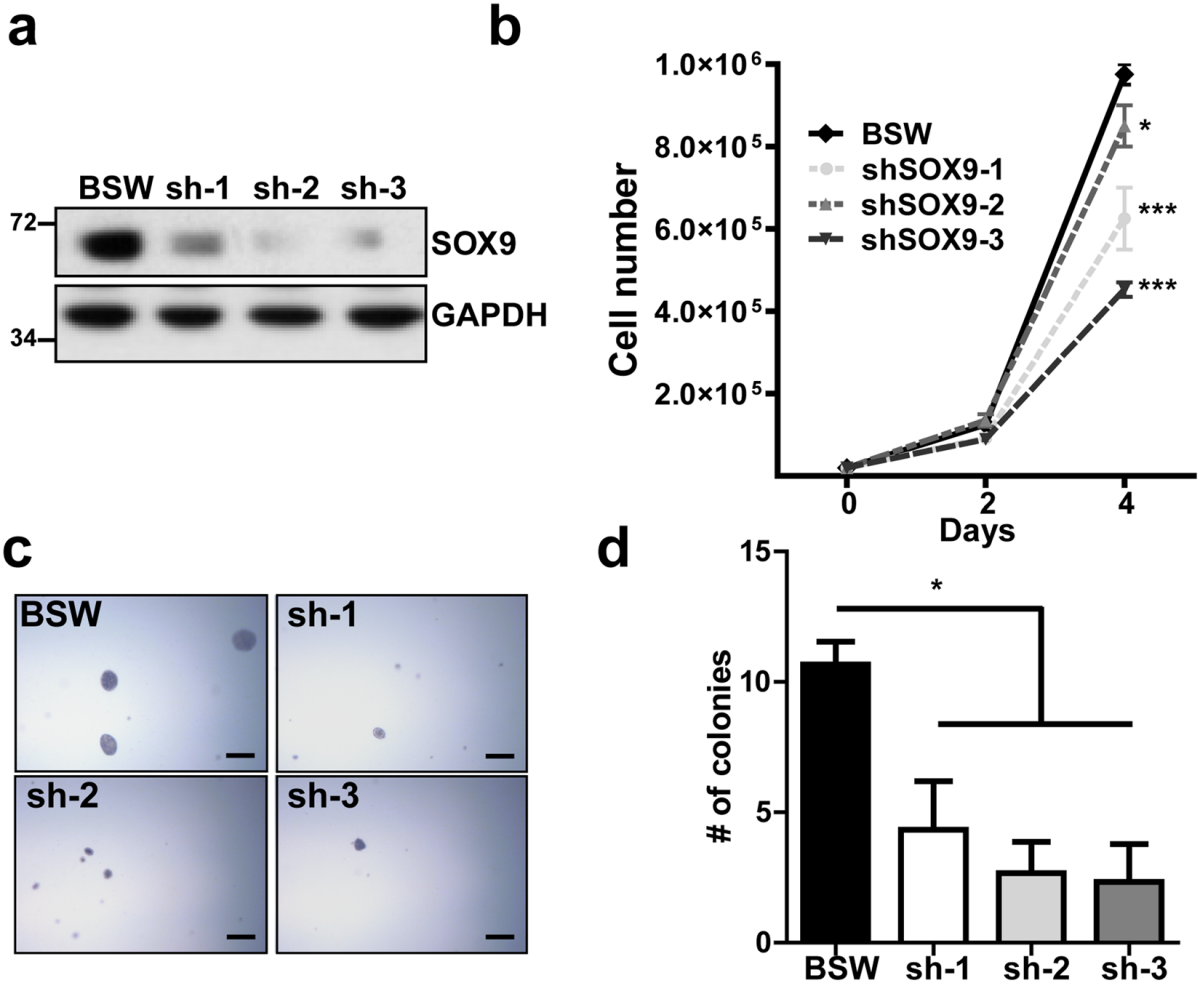
SOX9 knockdown inhibits proliferative and colony-forming properties of BSW cells. (**a**) Immunoblot showing levels of SOX9 protein after knockdown generated by 3 different shRNAs (sh-1, sh-2, sh-3), empty vector was used as a control in BSW cells. (**b**) Rate of cell proliferation, p < 0.05, Bonferroni post hoc analysis following 2-way ANOVA. (**c**) Soft agar colony formation assay. Cells were cultured in soft agar for 2 weeks, scale bar −500 μm. (**d**) Quantification of the soft agar assay, colonies larger than 50 μm in diameter were counted, n = 3. Data presented as mean values ± SEM. Means are different according to Turkey post hoc analysis following ANOVA, p < 0.05.

**Figure 4:**
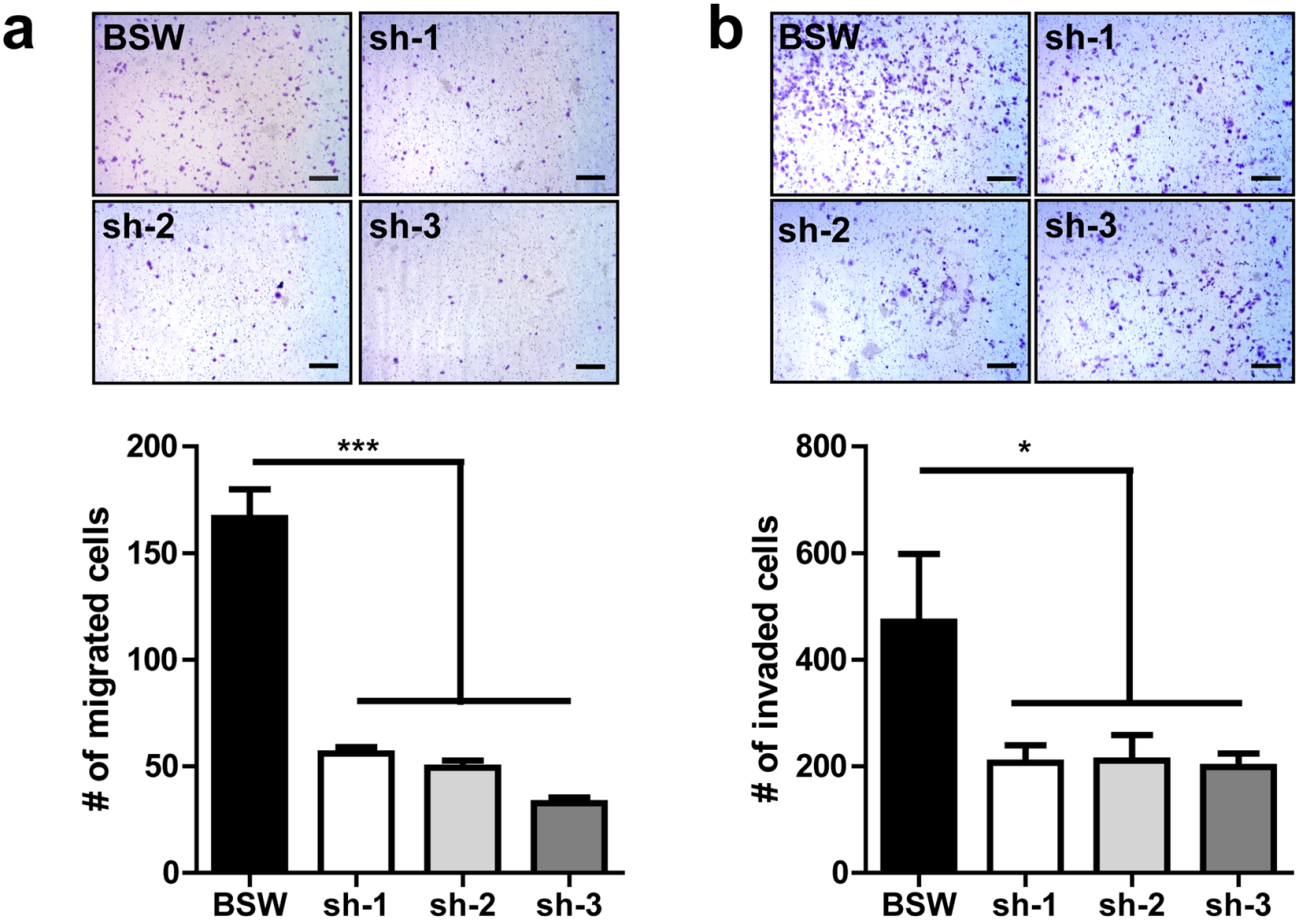
SOX9 knockdown attenuates migration and invasion of BSW cells *in vitro*. SOX9 knockdown was generated by 3 different shRNAs (sh-1, sh-2, sh-3), empty vector used as a control in BSW cells. BSW cells were added to control inserts (migration) or inserts coated with Matrigel (invasion) and incubated for 15 h. After removal of non-migrated and non-invaded cells, cells were fixed and stained with crystal violet and counted under a microscope. (**a**) Transwell migration assay. Bottom – quantification. (**b**) Transwell invasion assay. Bottom - quantification. Data presented as mean ± SEM, n = 10. Means are different according to Turkey post hoc analysis following ANOVA, p < 0.05.

To test our hypothesis, SOX9 expression in BSW cells was stably knocked down by shRNAs (Fig. 3a). The knockdown cells (shSOX9) exhibited a slower rate of proliferation (Fig. 3b) and formed substantially fewer colonies in the soft agar assay when compared to vector control cells (Fig. 3c,d). These results suggest that SOX9 depletion alleviates the survival of cells in matrix depleted conditions and potentially decreases their metastatic potential. *In vitro* migration and invasion assays further demonstrated a marked reduction in cell motility following SOX9 knockdown (Fig. 4). We also used an established NSCLC cell line H460 to compare the results of SOX9 downregulation in BSW cells to those in lung cancer cells. SOX9 knockdown also attenuated colony formation and decreased the proliferation rate of H460 cells (Supplementary Fig. S3), which is consistent with previous reports^28, 32^. Taken together, our results support the critical role of SOX9 overexpression in the malignant phenotype of SWCNT-exposed cells. Specifically, SOX9 knockdown reduced cell proliferation, colony formation, migration, and invasion - properties that all are considered as hallmarks of cancer^33^.

### SOX9 regulates SWCNT-induced cancer stem cells

**Figure 5:**
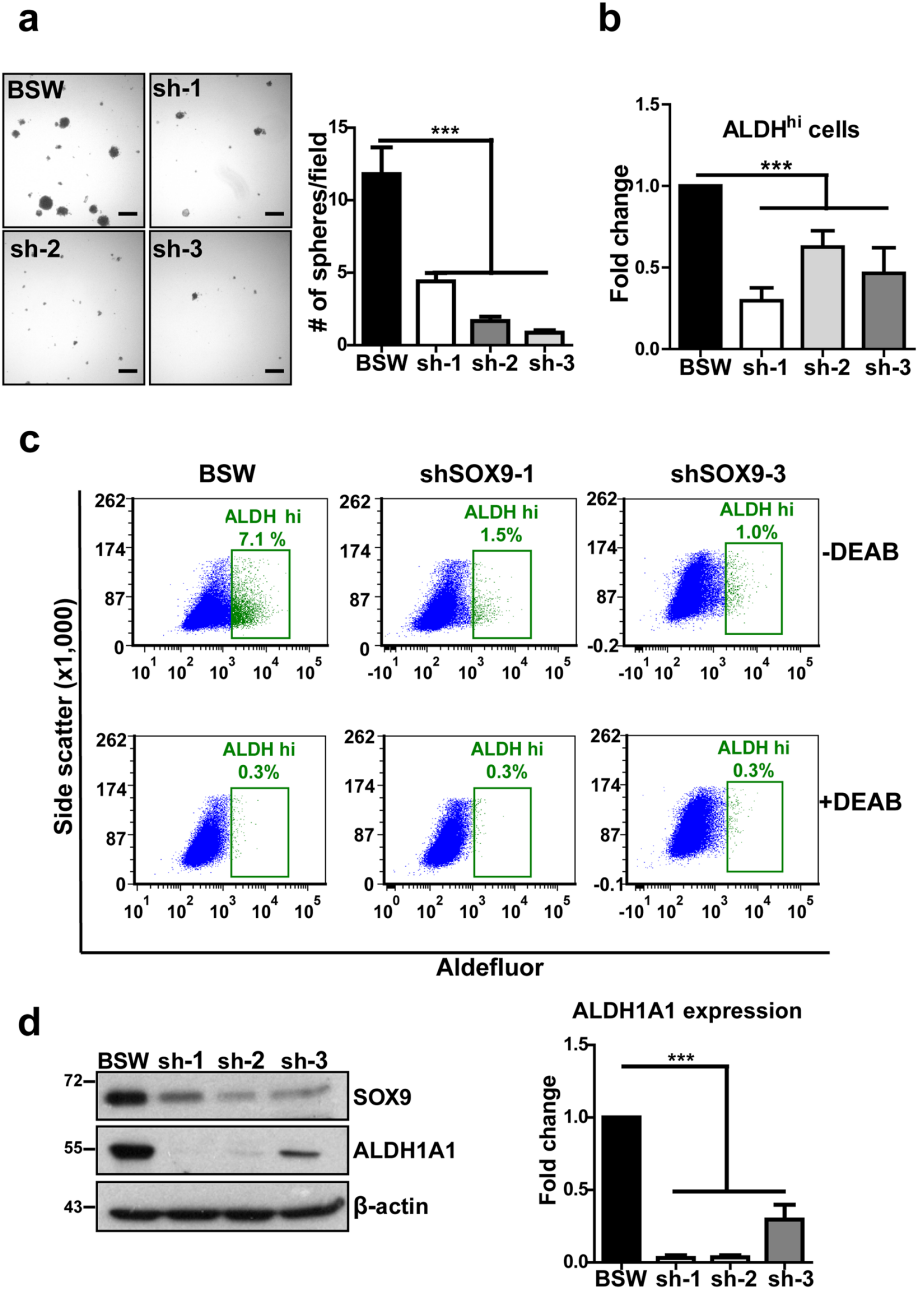
SOX9 regulates cancer stem cells in SWCNT-exposed cells. SOX9 knockdown was generated by 3 different shRNAs (sh-1, sh-2, sh-3), empty vector used as a control in BSW cells. (**a**) Tumor sphere formation assay. Right – quantitative analysis of tumor sphere formation, n = 15. (**b**) Fold change in the number of ALDH^hi^ cells in SOX9 knockdown over control BSW cells, 4 independent experiments combined (n = 4–9) measured by Aldefluor assay. (**c**) Representative flow cytometry results for Aldefluor assay. Top row – cells stained with the Aldefluor substrate only, bottom – cells stained with the Aldefluor substrate in the presence of the specific ALDH inhibitor DEAB. (**d**) SOX9 knockdown leads to a depletion of cancer stem cell marker ALDH1A1, right – quantification of relative ALDH1A1 expression, fold changes over control BSW cells, n = 3 independent experiments. Results are mean ± SEM. Means are different according to Turkey post hoc analysis following ANOVA, p < 0.05. DEAB - N,N-diethylaminobenzaldehyde.

The ability for self-renewal is a key characteristic of stem cells^36, 37^. The tumor sphere formation assay has frequently been used as a functional *in vitro* test to evaluate the self-renewal ability of adult stem cells and cancer stem cells^38, 39^. To evaluate the sphere forming capability of SWCNT-transformed cells and its regulation by SOX9, 1,000 control and shSOX9 cells were cultured under non-adherent conditions in serum-free medium. After 2 weeks in culture, the number of tumor spheres exceeding 50 µm in diameter was quantified. We found that SOX9 knockdown substantially inhibited sphere formation (Fig. 5a), suggesting that SOX9 positively regulates stem cells in our model.

Next, we evaluated the expression and activity of the common cancer stem marker ALDH, which is highly active in cancer stem cells and is frequently associated with poor clinical outcomes^23, 40, 41^. The Aldefluor assay was used to quantify ALDH activity in BSW and BSW-shSOX9 cells. The activated Aldefluor reagent is a cell-permeable fluorescent substrate for ALDH that accumulates inside cells after interaction with ALDH and can be subsequently detected using flow cytometry. Diethylaminobenzaldehyde (DEAB), a specific ALDH inhibitor, was used to evaluate background fluorescence and set up gates for flow cytometry. We found that ALDH activity was substantially decreased in SOX9 knockdown cells, as indicated by a reduced number of cells exhibiting high ALDH activity (ALDH^hi^ cells) (Fig. 5b,c). Consistent with the pattern of ALDH activity, expression levels of ALDH1A1, one of the main CSC-associated ALDH isoforms^42, 43^, were dramatically reduced following SOX9 knockdown (Fig. 5d). ALDH1A1 expression was also depleted in H460-shSOX9 cells (Supplementary Fig. S3), indicating that a correlation between SOX9 and ALDH expression is not limited to one specific cell system. These results suggest that SOX9 controls CSCs in BSW cells and possibly other cancer cell types through ALDH1A1.

### SOX9 knockdown attenuates BSW metastasis *in vivo*

**Figure 6:**
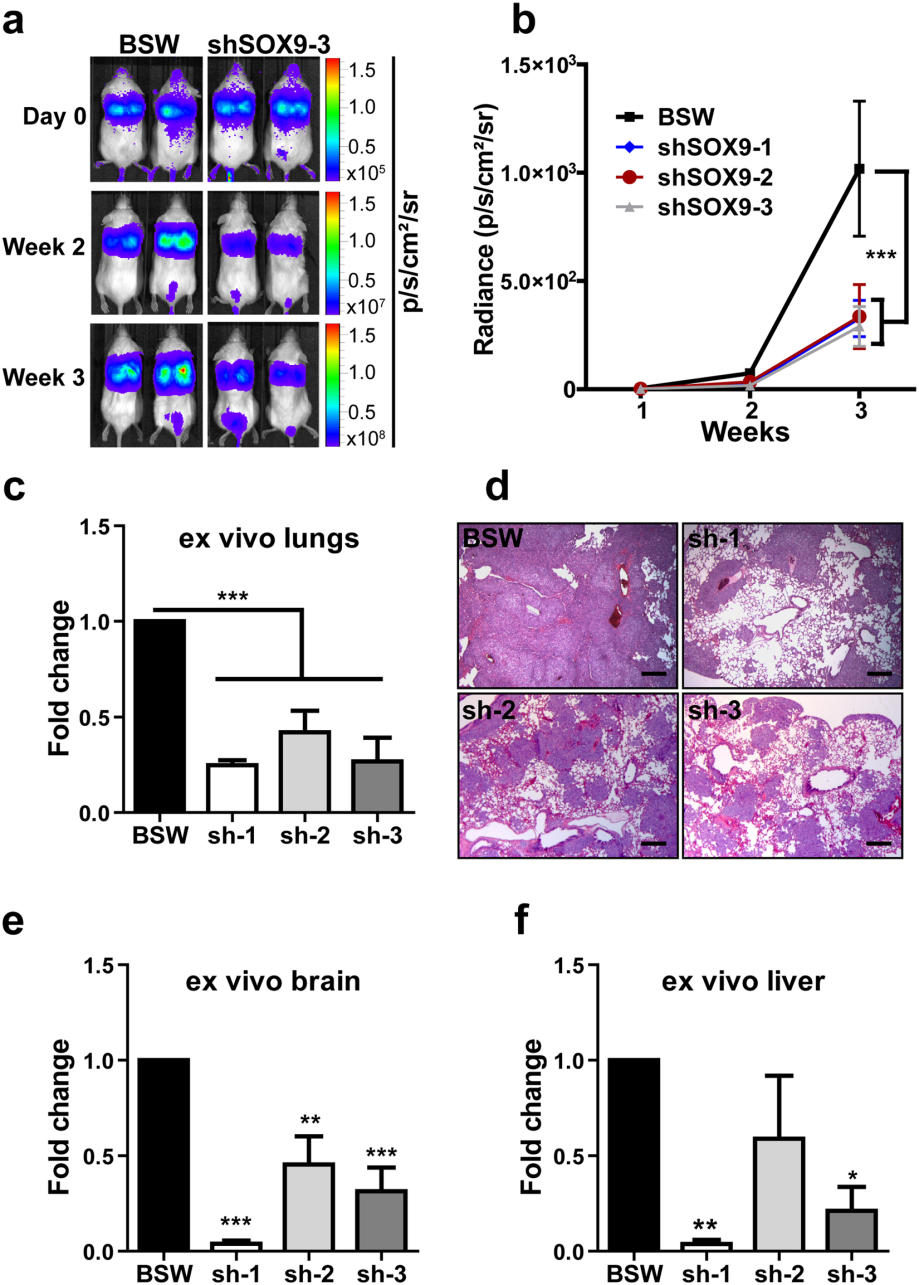
SOX9 knockdown inhibits BSW metastasis *in vivo*. Luciferase-labeled cells were injected intravenously via tail vein into NOD/SCID gamma mice (n = 4 per group) and mice were imaged right after cell injection and weekly thereafter. SOX9 knockdown was generated by 3 different shRNAs (sh-1, sh-2, sh-3), empty vector used as a control in BSW cells. (**a**) Representative whole body bioluminescence of lung tumors in mice injected with control vector (BSW) or SOX9 knockdown (shSOX9-3). (**b**) Time course of lung tumor growth generated by weekly whole body imaging, each time point normalized to the signal from day 0. Means are different according to Bonferroni post hoc analysis following 2-way ANOVA, p < 0.05. (**c**,e,**f**) *Ex vivo* luciferase signal from isolated mouse lungs, brain and liver, respectively. Means are different according to Turkey post hoc analysis following ANOVA. (**d**) Representative pictures of H&E stained lungs showing a notable decrease in tumor lung colonization by SOX9 knockdown cells comparing to control cells, scale bar −250 µm. Data are shown as mean ± SEM, n = 4.

We next evaluated whether SOX9 depletion affects the metastatic potential of BSW cells *in vivo*. Luciferase-labeled BSW and BSW-shSOX9 cells were intravenously injected into mice followed by weekly bioluminescence imaging. At the end of the experiment, mice were euthanized; their internal organs (brain, liver, spleen, pancreas, kidneys and lungs) were removed and imaged to evaluate metastatic lesions. Figure 6a shows representative whole-body bioluminescence images of mice bearing either the control or shSOX9 cells. Importantly, lung colonization by shSOX9 cells was dramatically decreased when compared to control cells (Fig. 6b). This was also confirmed by *ex vivo* lung imaging (Fig. 6c) and by histological analysis (Fig. 6d). The luminescent signal from distant metastases in the liver and brain also decreased (Fig. 6e,f). In addition, we observed an occasional luciferase signal from the kidneys and pancreas in the control but not in the shSOX9 animals (data not shown). Our findings indicate a significant role of SOX9 in the regulation of the metastatic properties of malignantly transformed BSW cells.

### SOX9 overexpression in normal Beas-2B cells induces stem cell-like properties

**Figure 7:**
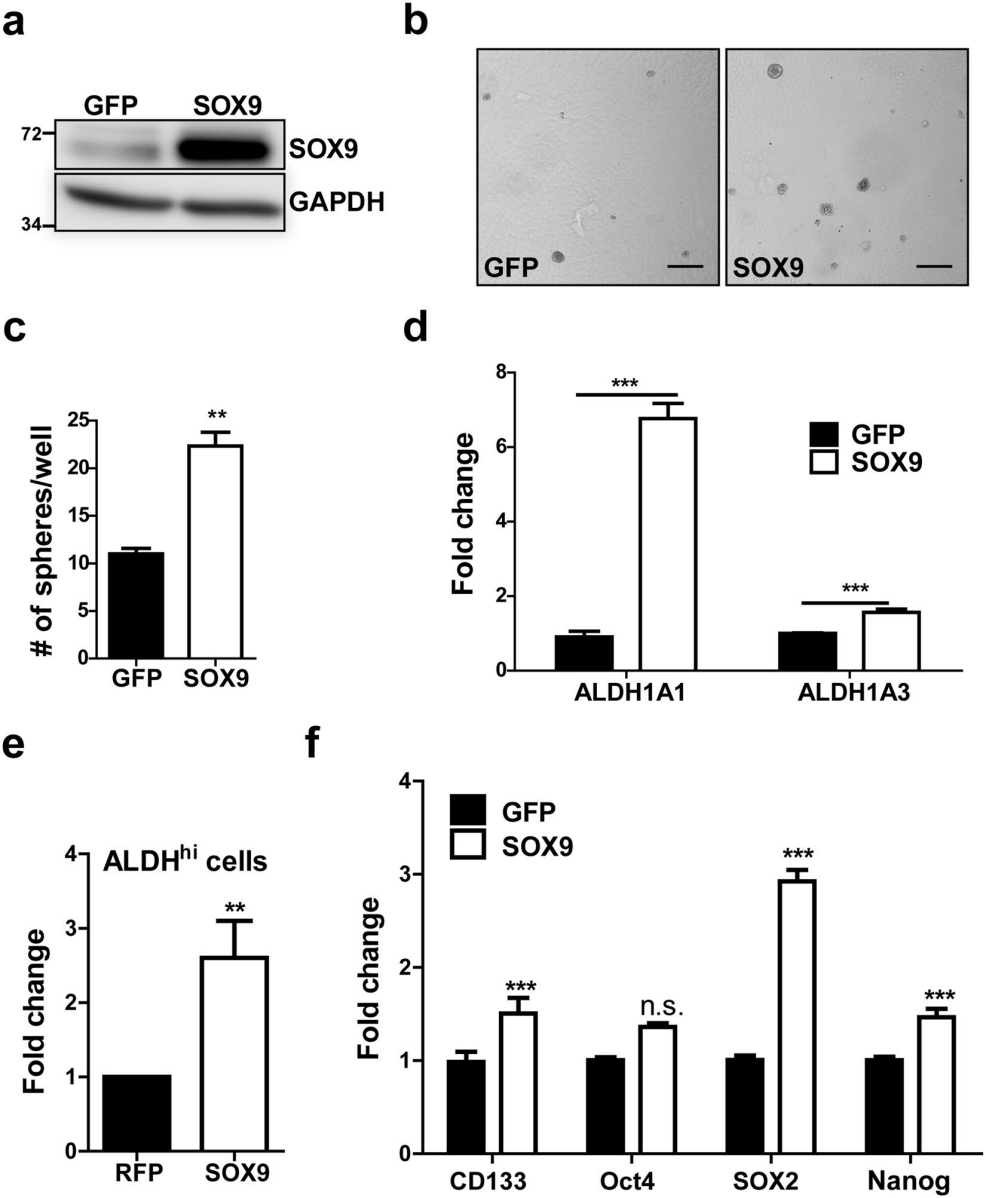
SOX9 overexpression in Beas-2B cells induces stem cell-like properties. (**a**) Level of SOX9 overexpression. (**b**) Representative images of tumor sphere formation. Scale bar, 200 µm. (**c**) Quantitative analysis of tumor sphere formation form (b), n = 3, p = 0.0019, two-tailed unpaired t-test. (**d**) RT-qPCR analysis of 2 ALDH isoforms, n = 6–12, 2 independent experiments combined, p < 0.05 by two-tailed unpaired t-test. (**e**) Fold change in the number of ALDH^hi^ cells in SOX9 overexpressing cells over control measured by Aldefluor assay, combined results from 4 independent experiments (n = 17), p = 0.004, two-tailed paired t-test. (**f**) Expression levels of stem cell markers were examined by RT-qPCR, n = 6–9, p < 0.05 according to Bonferroni post hoc analysis following 2-way ANOVA, GFP control vs SOX9 overexpression. n.s. - not significant. Data are shown as mean ± SEM.

We next overexpressed SOX9 in passage-matched control (B2B) cells to test whether this would be sufficient to recapitulate the phenotype of BSW cells. Of note, we achieved a maximum 5-fold increase of SOX9 expression (Fig. 7a), while BSW cells have on average a 15-fold increase in SOX9 expression in comparison to Beas-2B cells (Fig. 2a). Nevertheless, a 5-fold SOX9 overexpression in Beas-2B cells was sufficient to promote tumor sphere formation (Fig. 7b,c). Note that these cells normally form a small number of loose cell aggregates under low-attachment conditions, while SOX9 overexpression triggered the appearance of typical, round tumor spheres. Furthermore, mRNA levels of main NSCLC-associated isoforms ALDH1A1 and ALDH1A3^44^ as well as ALDH activity were upregulated by SOX9 (Fig. 7d,e). We also evaluated changes in the expression levels of stem cell related proteins in response to SOX9 overexpression. We observed an increased expression of CD133, a marker of lung CSCs^45^, and embryonic stem cell factors Nanog, SOX2 and Oct4, although the increase was not statistically significant in the case of Oct4 (Fig. 7f). Together, these results support the regulatory role of SOX9 in lung CSCs induction and ALDH1A1 expression.

## Discussion

In this study, we demonstrated that lung epithelial cells chronically exposed to SWCNTs may undergo malignant transformation and gain metastatic properties. These cells displayed traits typically characteristic of cancer cells, including anchorage-independent growth and *in vivo* tumor formation, supporting the previously published work^14, 19^. In addition, the metastatic potential of SWCNT-exposed cells was demonstrated for the first time. We observed that BSW cells can metastasize in both subcutaneous (Fig. 1) and tail vein mouse models (Fig. 6). We showed for the first time that SOX9 is a critical player in SWCNT-induced carcinogenesis, and that SOX9 depletion significantly reduces metastatic potential both *in vitro* (Fig. 4) and *in vivo* (Fig. 6).

Cancer stem cells (CSCs) have been considered the main source of cancer initiation, dissemination, and recurrence based on their ability for self-renewal, drug- and apoptosis resistance. Furthermore, multiple studies have illustrated that tumor initiating cells share some characteristics with regular stem cells and express stem cell markers. Thus, it is not surprising that many proteins that are involved in embryonic development, such as the SOX family of proteins, appear to be CSC drivers. Our study demonstrated a high level of SOX9 expression in aggressive SWCNT-exposed cells, consistent with recent reports of SOX9 up-regulation in multiple cancer types^26^. However, the functional role of SOX9 in transformed cells required further investigation. We hypothesized that the elevated SOX9 expression in cancer cells and SWCNT-exposed cells may induce CSC formation, which in turn could drive tumor formation and metastasis. Indeed, we found that SOX9 knockdown strongly inhibited tumor sphere formation and anchorage-independent growth (Figs 5a and 3c,d). Bioluminescence signals from mouse lungs in the tail vein model clearly demonstrated that SOX9 knockdown decreases lung colonization (Fig. 6). Together, these results suggest that SOX9 regulates cancer cell survival, consistent with our previous work in lung cancer^32^.

We tested how SOX9 affects CSC markers to further evaluate our hypothesis. Elevated activity of ALDH, an enzyme with a variety of functions including detoxification, is associated with poor clinical outcomes due to metastasis and has been linked to CSCs^23, 40, 41^. We observed a substantial decrease in ALDH activity following SOX9 knockdown (Fig. 5d,c), suggesting that ALDH may be a potential downstream target of SOX9. Likewise, the expression of ALDH1A1, an ALDH isoform most commonly associated with the CSC activity^23, 40, 42^, was dramatically reduced in both BSW and H460 knockdown cells (Fig. 5d, Supplementary Fig. S3). Notably, ALDH serves not only as a stem cell marker, but also governs stem cell differentiation^46, 47^ by producing retinoic acid^48^. To further explore the potential relationship between ALDH and SOX9, we overexpressed SOX9 in control Beas-2B cells. We observed an increase in tumor sphere formation (Fig. 7b,c), which is consistent with the knockdown experiments. Likewise, mRNA levels of the reported to be overexpressed in NSCLC ALDH isoforms ALDH1A1 and ALDH1A3^44^ were elevated, validating our findings in SOX9 knockdown cells. We also observed an increased expression of stem cell makers CD133, Nanog and SOX2 (Fig. 7f), suggesting that SOX9 overexpression renders cells less differentiated. Collectively, our observations strongly support the positive regulatory role of SOX9 in CSC formation, consistent with other studies in pancreatic, esophageal, and colorectal cancers^30, 49, 50^. To our knowledge, the present study demonstrates the link between SOX9 and ALDH expression for the first time. This provides a key mechanism underlying CSC induction by SOX9, while the exact mode of ALDH upregulation remains a subject for further investigation.

A limitation of this study is that the results were largely derived from one cell model (Beas-2B cells) and one type of CNTs. However, our group observed a similar neoplastic-like transformation in several other models and with different types of CNTs. For examples, MWCNTs have recently been shown to induce neoplastic transformation of primary human small airway epithelial cells^51^. Both SWCNTs and MWCNTs similarly induced such transformation in hTERT-immortalized human small airway epithelial cells after a long-term exposure^15^. The induction of fibroblast stem-like cells and ALDH1A1 expression was also reported in primary human lung fibroblasts exposed to SWCNTs or MWCNTs^51^. Furthermore, co-culture of these activated fibroblasts with lung cancer cells promoted CSC-related properties and tumor formation by cancer cells^52^. Of note, animal experiments in the current study were conducted in immunodeficient mice, and therefore these results should be interpreted with caution. Nonetheless, several other studies using immunocompetent mice and rats consistently indicate the tumorigenic potential of SWCNTs and MWCNTs^9–12^. While SOX9 is implicated as a key regulator of SWCNT-induced carcinogenesis in this report, other regulatory mechanisms are likely to be involved due to the complexity of carcinogenic process. For example, Shvedova *et al*. showed that inhalation or aspiration of SWCNTs caused lung inflammation and fibrosis along with K-ras mutation in immunocompetent mice^53^. Since K-ras is a known oncogene, whose mutational activation is frequently associated with an increased risk of lung cancer, it is quite possible that such mutation may be involved in SWCNT-induced carcinogenesis, although this has not been demonstrated yet. In addition, several studies evaluating CNT carcinogenicity via whole transcriptome arrays have identified cancer signatures and known cancer prognostic markers in exposed mouse lung tissues^54, 55^. Some of the identified genes such as caveolin-1 and Bcl-2 have already been implicated in CNT-induced carcinogenesis^20, 56^ and may be responsible for the early neoplastic transformation induced by SWCNTs.

Lung cancer is a progressive disease commonly associated with a long-term exposure to carcinogens. However, prolonged exposure studies to nanomaterials are lacking and are greatly needed for risk assessment and for safe-by-design strategies. Our results suggest that long-term exposure to SWCNTs could transform normal epithelial cells into metastatic tumor cells. Although multiple mechanisms and signaling pathways are likely to be involved in the transformation process, we report here the critical role of SOX9 in the transformation through CSC-related mechanisms. Given that CSCs may be involved in cancer initiation steps, we suggest that SOX9 may be used as an early biomarker for SWCNT-induced carcinogenesis. It remains to be elucidated whether SOX9 up-regulation and its contribution to malignancies are specific to SWCNTs or could be extrapolated to other CNTs. Such information will aid in the design of nanomaterial-specific biomarkers for risk assessment and safe-by-design efforts.

## Methods

### Cell culture and exposure to SWCNTs

Immortalized human bronchial epithelial Beas-2B cells were obtained from the American Type Culture Collection (ATCC). Cells were cultured in advanced Dulbecco’s modified Eagle medium (DMEM) (Life Technologies) supplemented with 1% fetal bovine serum (FBS, Atlanta Biologicals), 2 mM L-glutamine, 20 mM HEPES, 100 units/mL penicillin and 100 μg/mL streptomycin in 5% CO_2_ at 37 °C. This cell model has been reported to be an appropriate model for *in vitro* lung carcinogenesis studies^57^. The cells were exposed to well-characterized single-walled CNTs (SWCNTs), as previously described^14, 19^. Briefly, SWCNTs (Carbon Nanotechnology (CNI)) were purified by acid treatment to remove metal contaminates. Particle characterization studies were performed at NIOSH research facilities, and the results are summarized in Table 1. Particles were treated with acetone and placed in an ultrasonic bath at room temperature for 24 h. The dispersed CNTs were then filtered from the solution using a 20 μm nylon mesh screen followed by a 0.2 μm polytetrafluoroethylene (PTFE) filter. After filter collection, the dispersed CNTs were washed thoroughly with distilled water to remove acetone. The filter was dried overnight in vacuum and weighed to determine the quantity of SWCNTs. The particles were suspended in phosphate-buffered saline (PBS) by brief sonication and added to the cells every 3–4 days when changing the culture medium. Subconfluent cultures of Beas-2B cells were continuously exposed to a low-dose, occupationally relevant concentration (0.02 μg/cm^2^ or 0.1 μg/mL) of SWCNTs in culture for 6 months and passaged weekly. Cells were rinsed with PBS prior to culture medium changes and cell passaging to reduce potential SWCNT bioaccumulation over the exposure period.

The dose of SWCNTs used in this study was calculated based on reported effects of *in vivo* MWCNT exposure, normalized to mouse alveolar surface area. The lowest dose, which induced a biological response *in vivo*, was 10 μg/mouse lung (0.5 mg/kg body weight)^6^. Dividing this dose by the average mouse alveolar surface area (~500 cm^2^) gives the *in vitro* surface area dose of 0.02 μg/cm^2^, which is roughly equivalent to a human lung burden for 8 hours/day over a month at 400 μg/m^3^ (high CNT level reported in a research facility)^58^ or about 3 years at 10 μg/m^3^ (average CNT level in U.S. facilities)^59^. The cells were cultured in normal medium without SWCNTs for at least ten passages prior to further experiments Control cells were cultured for the same period of time in the above described culture medium. H460 (NCI-H460) cells were purchased from the ATCC and were passaged less than 20 times. H460 cells were maintained in RPMI medium (Corning) supplemented with 10% FBS, 100 units/mL penicillin, and 100 μg/mL streptomycin.

### Plasmids and generation of stable cell lines

pLKO.1 lentiviral plasmids for SOX9 knockdown generation were purchased from GE Healthcare (#RHS4533-EG6662). An empty vector was used as a control, and the virus was produced in HEK293T cells (ATCC). Briefly, HEK293T cells were co-transfected with the shRNA containing pLKO.1 plasmids or empty pLKO.1 vector, pCMV-VSV-G envelope, and psPAX2 packaging plasmids (Addgene #8454, 12260) in the presence of FuGene 6 transfection reagent (Promega). The medium containing viral particles was collected at 24, 36, and 48 hours post-transfection, pooled, and used to infect SWCNT-exposed (BSW) cells in the presence of hexadimethrine bromide (Sigma-Aldrich) at a final concentration of 5 μg/mL. After infection, cells were selected with 1 μg/mL of puromycin (Life Technologies). For SOX9 overexpression, SOX9 cDNA from pCMV-AC-GFP-SOX9 (Origene) was amplified by PCR introducing BamHI/SalI restriction sites and was subcloned into pLenti CMV GFP Zeo (Addgene #17449), replacing GFP. pLenti CMV GFP Zeo vector was used as a control. For flow cytometry experiments (Aldefluor assay), control vector with RFP instead of GFP was used. Viral particles were produced as described above.

### Immunoblotting

Cells were lysed in a non-reducing loading buffer containing 63 mM Tris-HCl pH 6.8, 10% glycerol, and 2% sodium dodecyl sulfate (SDS). Protein concentrations were quantified using the Pierce BCA protein assay kit (Thermo-Scientific). Proteins (40 μg) were resolved under denaturing conditions by 7.5–12% SDS-polyacrylamide gel electrophoresis (SDS-PAGE) and transferred onto PVDF membranes (EMD Millipore). Membranes were blocked in 5% non-fat milk in TBS-T buffer (25 mM Tris-HCl, pH 7.4, 125 mM NaCl, and 0.1% Tween 20) for 1 hour, washed 3 times for 5 minutes with TBS-T buffer, and incubated with appropriate primary antibodies at 4 °C overnight. Membranes were rinsed 3 times for 5 minutes with TBST-T buffer; membranes were then incubated with appropriate HRP-conjugated secondary antibodies (Jackson Immunoresearch), diluted in TBS-T for 1 hour at room temperature. Immune complexes were detected by enhanced chemiluminescence (Pierce ECL, Thermo-Scientific; Immobilon HRP Substrate, Millipore) and quantified with the Image Studio Lite program (Li-COR Biosciences). Antibodies used in this study are listed in the Supplementary Table 1. Full length unprocessed blot images are provided in the Supplementary Materials.

### Flow cytometry

The ALDH^hi^ population (cells with high aldehyde dehydrogenase activity) was identified by staining cells with the Aldefluor kit (Stem Cell Technologies), according to the manufacturer’s instructions. Briefly, for each sample, 3 × 10^5^ cells were incubated in the Aldefluor assay buffer with the activated Aldefluor substrate for 45 minutes at 37 °C in the presence or absence of the specific ALDH inhibitor diethylaminobenzaldehyde (DEAB). Tubes stained with both the Aldefluor substrate and DEAB served as a negative control for each sample. ALDH converts Aldefluor substrate into a fluorescent product, and cells with high ALDH activity (ALDH^hi^) were detected by BD Fortessa cell analyzer (BD Biosciences). The flow cytometry gates were set to obtain 0.1% ALDH^hi^ cells in substrate + inhibitor tubes for each sample. All experiments were performed at least 3 times.

### Soft agar colony formation assay

Cells were suspended in a soft agar medium containing 0.33% Difco agar (BD Biosciences) in 2x EMEM medium (Lonza) supplemented with L-glutamine, antibiotics and 15% FBS, and plated onto 6-well plates over a layer of 0.5% soft agar medium. Cells were cultured under standard cell culture conditions and were fed with 0.2 mL of the regular medium twice a week. Plates were observed under a light microscope after 2 weeks, and colonies exceeding 50 μm in diameter were scored. Experiments were performed in triplicates; at least 5 fields of view for each replicate were evaluated.

### Migration and invasion assays

Cells were seeded in the upper chamber of Transwell matrigel coated (invasion) or control inserts (migration) with 8 µm pores (Corning) in serum-free medium and were allowed to migrate or invade toward the serum-containing bottom chamber for 15 hours. Cells were subsequently removed from the top part of the chamber using a cotton swab, and cells on the bottom side were stained with crystal violet. Experiments were performed in at least duplicates, 5 random fields were photographed under a light microscope for each replicate.

### Cell proliferation assay

The rate of cell proliferation was measured by seeding equal numbers of cells onto 6-well plates in duplicates and counting cells with the Countess automated cell counter (Life Technologies) every 2 days until plates become confluent.

### Tumor sphere formation assay

Cells were plated onto ultra-low attachment 24-well plates (Corning) in 0.8% methylcellulose (MC)-based serum-free medium (Stem Cell Technologies, #H4100) supplemented with 20 ng/mL epidermal growth factor (BD Biosciences), 10 ng/mL basic fibroblast growth factor (Sigma-Aldrich), and 5 mg/mL insulin (Sigma-Aldrich). The number of tumor spheres exceeding 50 µm in diameter (20 µm for Beas-2B cells) was quantified under a light microscope after 2 weeks of culture under standard cell culture conditions. All experiments were performed in triplicates and repeated at least twice, with 5 fields of view analyzed for each replicate.

### Mouse xenograft experiments

All experiments were performed in accordance with the Guidelines for Animal Experiments at West Virginia University and approved by the Institutional Animal Care and Use Committee. Immunodeficient NOD/SCID gamma mice (NSG), strain NOD.Cg-Prkdc^scid^ Il2rg^tm1Wjl^/SzJ (Jackson Laboratory), were injected with 1 × 10^6^ of luciferase-labeled cells subcutaneously or via the tail vein. Tumor growth was monitored weekly using IVIS Lumina II *in Vivo* Imaging system (PerkinElmer), and an external caliper (VWR International) was used for the subcutaneous model. Tumor volume was calculated using the formula: tumor volume [mm^3^] = 1/2 (length [mm]) × (width [mm]^2^). At the end of the experiments mice were euthanized, and their organs were removed and imaged *ex vivo* on a Petri dish to evaluate metastasis. Bioluminescent images were quantified using the Living Image software (PerkinElmer). Tissue processing and H&E staining was carried out by the WVU Pathology Laboratory for Translational Medicine using standard procedures.

### Immunohistochemistry

Tissue sections were deparaffinized using standard techniques. Antigen retrieval was carried out by heating slides in a microwave in citrate buffer (10 mM citric acid, 0.05% Tween 20, pH 6.0) for 30 min. Samples were blocked for 30 min with normal goat serum (BioGenex, #HK112–9K) and incubated with primary antibodies overnight at 4 °C (specific antibodies used are listed in Supplementary Table 1). Slides were rinsed with TBS-T buffer 3 times and incubated with fluorescently labeled secondary antibodies (Life Technologies) for 1 hour at room temperature; after which they were rinsed with TBS-T buffer 3 times and mounted with the ProLong DAPI (Life Technologies). Cells were visualized with a Zeiss fluorescent microscope (Carl Zeiss).

### Immunofluorescence

Cells were grown overnight on 8-well chamber slides (Nunc), fixed with 4% paraformaldehyde and permeabilized with 100% methanol at −20 °C prior to blocking with 5% BSA in PBS. Cell were incubated with specific primary antibodies (listed in Supplementary Table 1), rinsed with PBS and incubated with secondary antibodies. Secondary antibodies were goat-anti-rabbit Alexa Fluor 546 (Life Technologies) and donkey-anti-mouse Alexa Fluor 488 (Jackson Immunoresearch). Slides were mounted with Fluoroshield with DAPI (Abcam) and visualized with a Zeiss fluorescent microscope (Carl Zeiss).

### RT-qPCR

Total RNA was purified using the RNeasy kit (Qiagen). Reverse transcription was performed with SuperScript III Reverse Transcriptase (Life Technologies) using oligo(dT) primers. Quantitative real-time PCR was carried out using the SYBR Green Master Mix (Applied Biosystems) in an ABI 7500 Real-Time PCR Cycler (Applied Biosystems). Results were calculated using the 2^−ΔΔCt^ method; glyceraldehyde-3-phosphate dehydrogenase (GAPDH) served as the internal control. Primer sequences are listed in the Supplementary Table 2.

### Statistical analysis

The data represent means ± SEM from two or more independent experiments as indicated. Statistical comparisons were made using two-tailed Student’s t test. When more than two groups were analyzed, ANOVA was used. Statistical analysis was performed with GraphPad Prism software (GraphPad), and p < 0.05 was considered statistically significant.

### Data Availability

The datasets generated during and/or analyzed during the current study are available from the corresponding author on reasonable request.

### Disclaimer

The findings and conclusions in this report are those of the authors and do not necessarily represent the views of the National Institute for Occupational Safety and Health.

## Electronic supplementary material

Supplementary Information
