# Supplementary material for: SOX9 Regulates Cancer Stem-Like Properties and Metastatic Potential of Single-Walled Carbon Nanotube-Exposed Cells
Source: Sci Rep. 2017 Sep 14;7:11653. doi: 10.1038/s41598-017-12037-8 (PMC5599589; doi:10.1038/s41598-017-12037-8)

Supplementary materials

SOX9 Regulates Cancer Stem-Like Properties and Metastatic Potential of Single-Walled Carbon Nanotube-Exposed Cells

Maria A. Voronkova[^], Sudjit Luanpitpong[‡,§], Liying Wang Rojanasakul[¶], Vincent Castranova[‡], Cerasela Zoica Dinu[||], Heimo Riedel[^#], and Yon Rojanasakul[*,^,‡]

[^]West Virginia University Cancer Institute, [‡]Department of Pharmaceutical Sciences, [||]Department of Chemical Engineering, [#]Department of Biochemistry, West Virginia University, WV 26506, United States; [§]Siriraj Center of Excellence for Stem Cell Research, Faculty of Medicine Siriraj Hospital, Mahidol University, Bangkok 10700, Thailand; [¶]Allergy and Clinical Immunology Branch, National Institute for Occupational Safety and Health, Morgantown, WV 26505, United States.

Corresponding Author

*Correspondence should be addressed to Yon Rojanasakul, West Virginia University, Morgantown, WV 26506, USA. Phone: +1 304 293 1476. Email: yrojan@hsc.wvu.edu

Figure S1

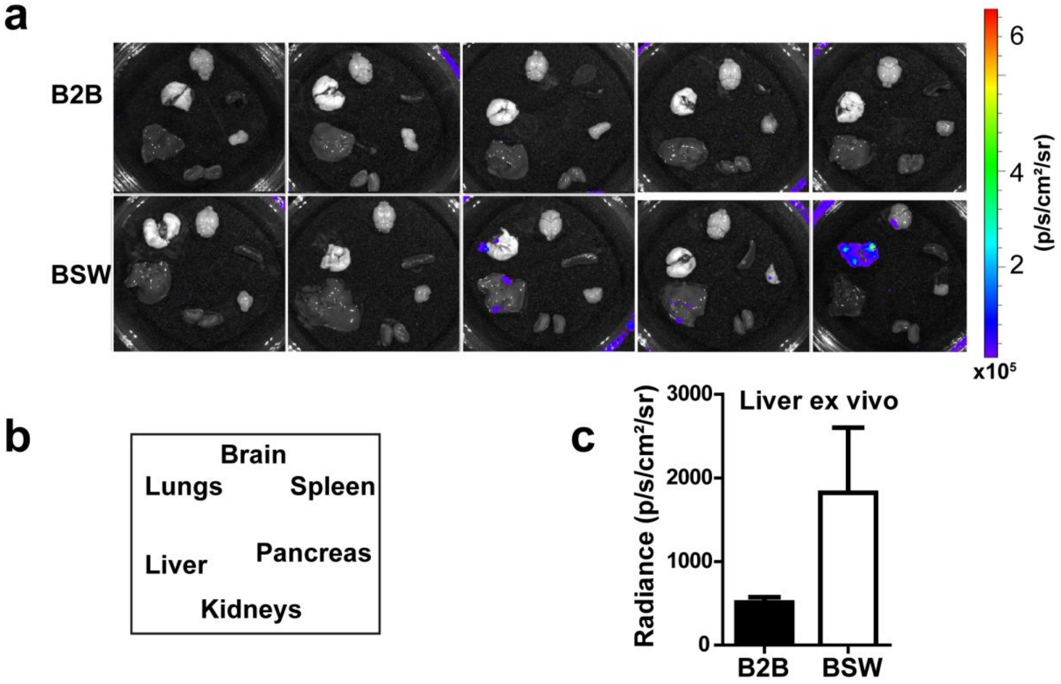

Supplementary Figure 1. BSW cells spontaneously metastasize in the subcutaneous mouse xenograft. Luciferase-labeled cells were injected subcutaneously into flanks of NOD/SCID gamma mice (n=5 per group). **(a)** *Ex vivo* bioluminescent signal from isolated mouse organs at the end of the experiment at week 4. **(b)** Schematic representation of organ positions in **a.** **(c)** *Ex vivo* signal from liver. Error bars are mean ± SEM.

μm.

Figure S2

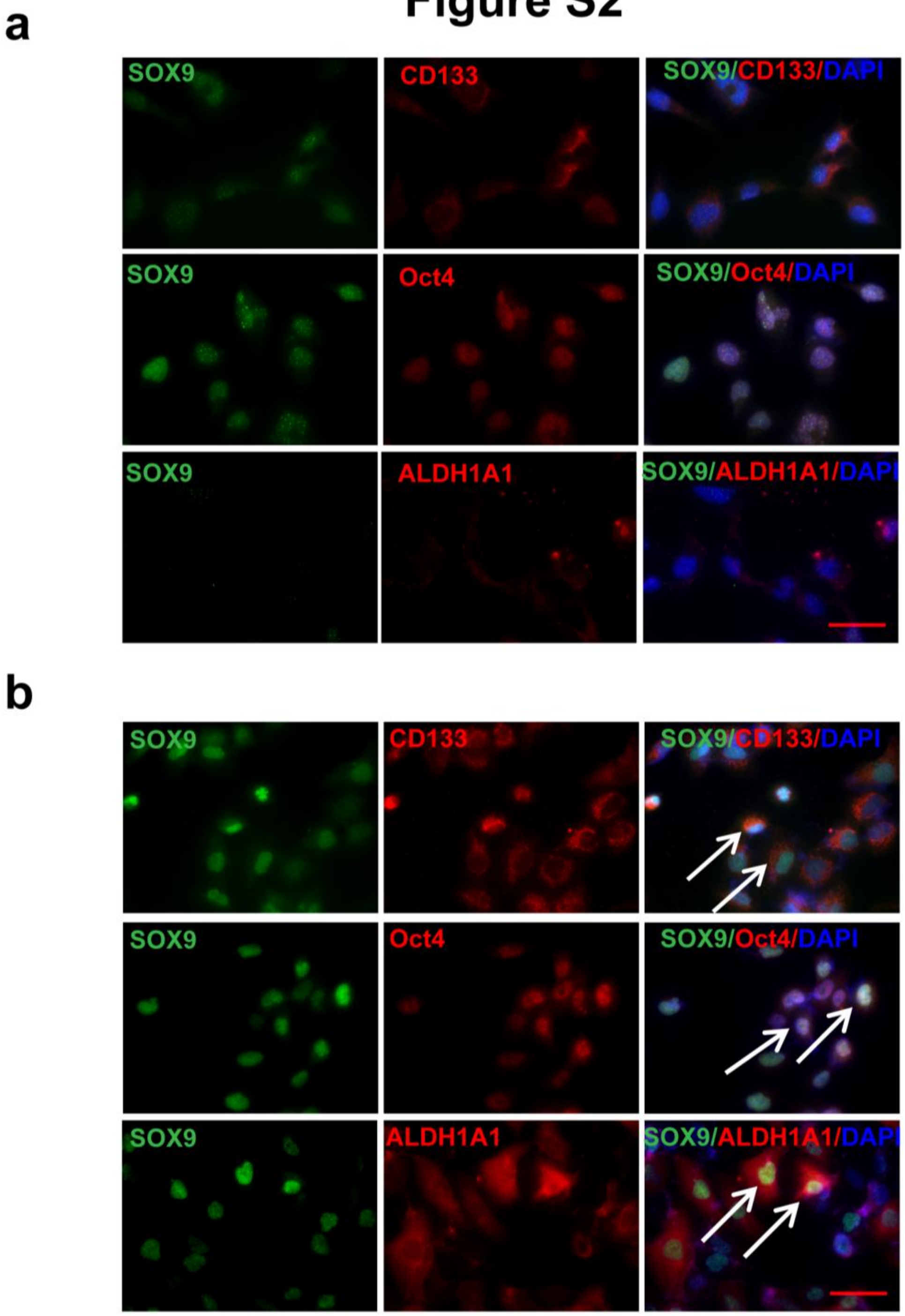

Supplementary Figure 2. SOX9 colocalizes with CSC markers. Immunofluorescence staining of CSC markers in SWCNT-treated and control cells. **(a)** Control Beas-2B cells, **(b)** BSW cells. Arrows specify cells expressing high levels of SOX9 and indicated CSC markers. Scale bar, 50 μm.

Figure S3

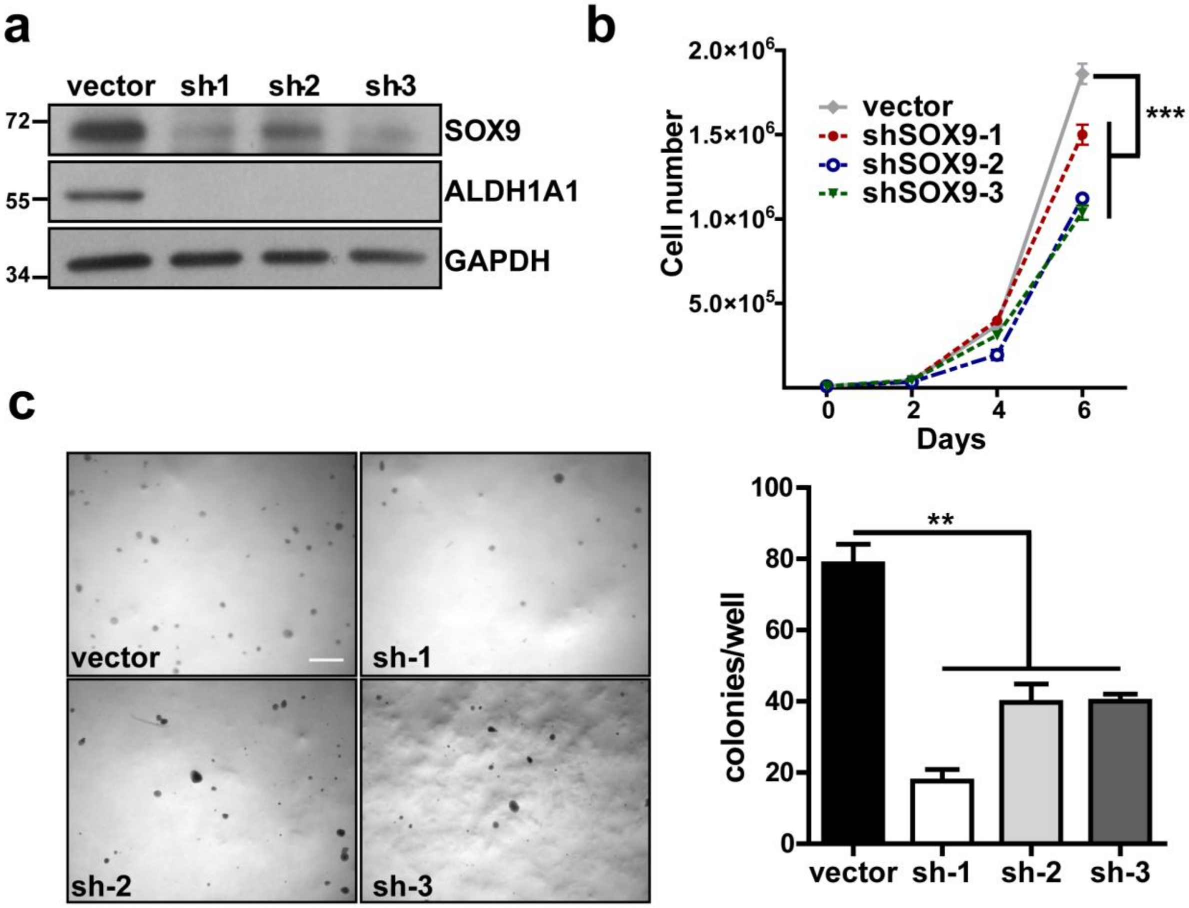

Supplementary Figure 3. SOX9 knockdown in H460 cells. (a) SOX9 knockdown by 3 shRNAs diminishes ALDH1A1 expression. (**b)** Cell proliferation assay, n = 2. P < 0.001, 2way repeated measures ANOVA followed by Bonferroni posttests, shRNAs vs vector control. (**c)** Soft agar colony formation. p < 0.05, Turkey post hoc analysis following ANOVA, n = 3. Scale bar, 2 mm.

Supplementary Table 1. List of antibodies used in this study.

Name	Company	Catalog No	Application
SOX9	EMD Millipore	AB5535	WB, IHC
SOX9	EMD Millipore	MABC785	IF
ALDH1A1	Abcam	ab52492	WB
ALDH1A1	EMD Millipore	MABN838	WB
ALDH1A1	Cell Signaling	54135	IF
β-actin	Sigma-Aldrich	A5441	WB
GAPDH	EMD Millipore	CB1001	WB
a-rabbit-HRP	Jackson ImmunoResearch	711-035-152	WB
a-mouse-HRP	Jackson ImmunoResearch	715-035-151	WB
Human mitochondria	EMD Millipore	MAB1273	IHC
CD133	Cell Signaling	86781	IF
Oct4	Abcam	ab19857	IF

WB – western blotting, IHC – immunohistochemistry, IF – immunofluorescence.

Supplementary Table 2. Oligonucleotides for real-time quantitative PCR analysis.

Name	Forward (5' to 3')	Reverse (5' to 3')
ALDH1A1	AGTGTGGGTGAATTGCTATGG	TGTGACTGTTTTGACCTCTGT
ALDH1A3	CTTCTGCCTTAGAGTCTGGAAC	TGCTACGTGGACAAGAACTG
GAPDH	CCCACTCCTCCACCTTTGAC	ATGAGGTCCACCACCCTGTT
CD133	TGAATAGCAACCCTGAACTGAG	TTGGCGTTGTACTCTGTCAG
Oct4	TCTTCAGGAGATATGCAAAGCAGA	GATCTGCTGCAGTGTGGGT
SOX2	CACTGCCCCTCTCACAC	TCCATGCTGTTTCTTACTCTCC
Nanog	GAAATACCTCAGCCTCCAGC	GCGTCACACCATTGCTATTC

Supplementary Figure 4. Full images of unedited western blots used in Figure 2a.

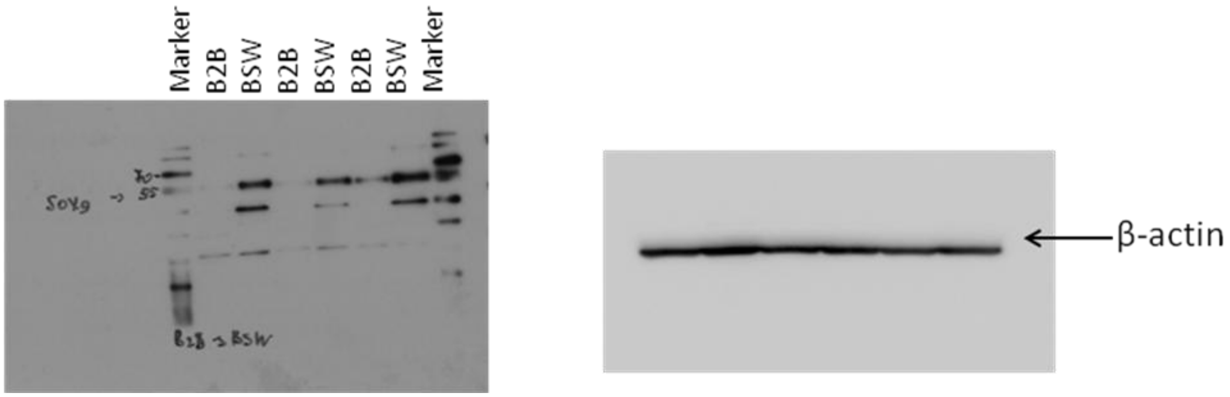

Same blot was reprobed with anti β-actin antibodies.

Supplementary Figure 5. Full images of unedited western blots used in Figure 3a.

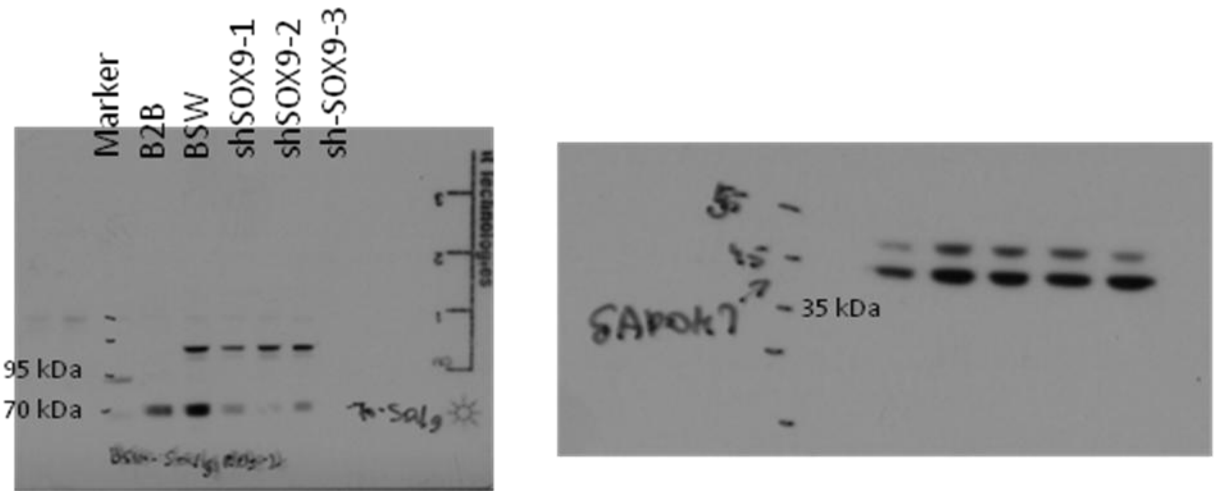

Same blot was reprobed with anti GAPDH antibodies.

Supplementary Figure 6. Full images of unedited western blots used in Figure 5d.

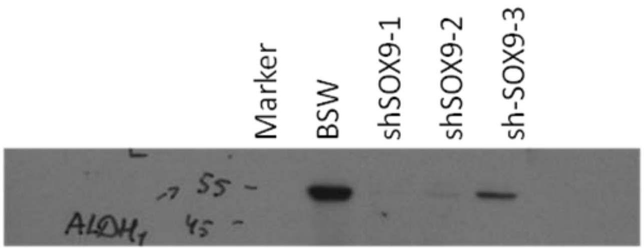

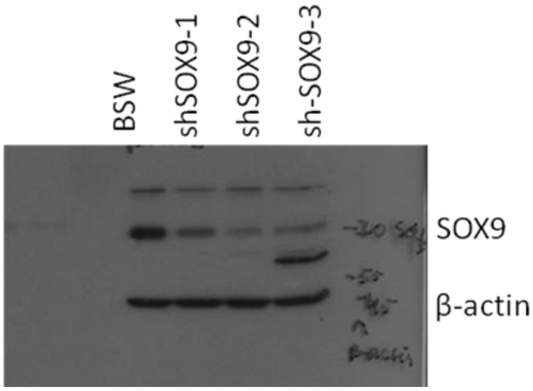

Supplementary Figure 7. Full images of unedited western blots used in Figure 7a.

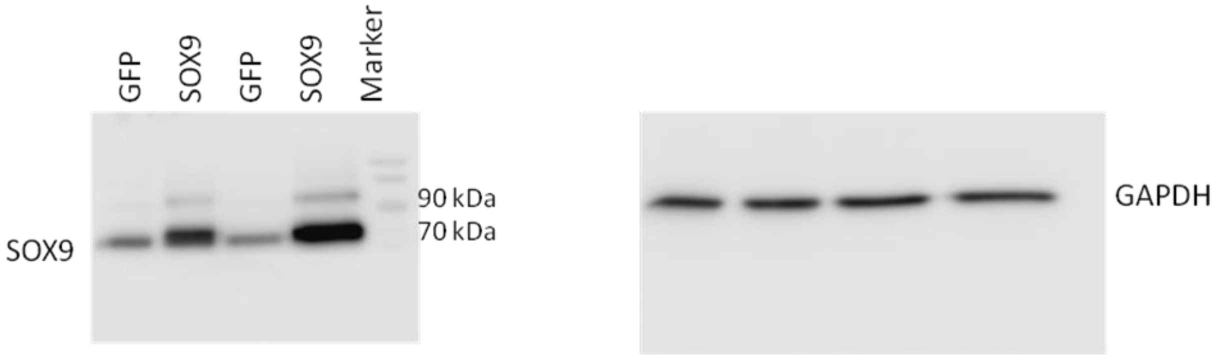

Same blot was reprobed with anti GAPDH antibodies.

Supplementary Figure 8. Full images of unedited western blots used in Figure S3.

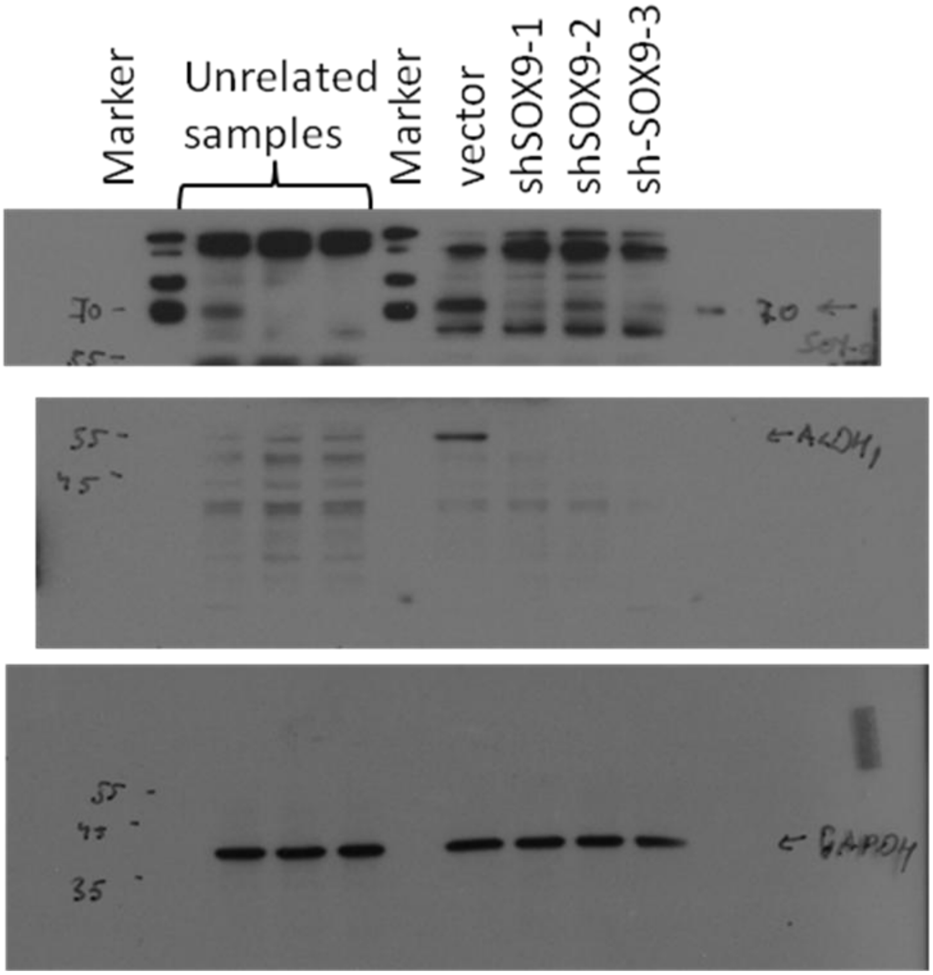

Supplement: Supplementary file 1 — Supplementary Information [file 41598_2017_12037_MOESM1_ESM.pdf]
